# The language profile in multiple system atrophy: an exploratory study

**DOI:** 10.1007/s00702-021-02372-6

**Published:** 2021-07-03

**Authors:** Sofia Cuoco, Marina Picillo, Immacolata Carotenuto, Roberto Erro, Eleonora Catricalà, Stefano Cappa, Maria Teresa Pellecchia, Paolo Barone

**Affiliations:** 1 Neuroscience Section, Department of Medicine, Surgery and Dentistry, Center for Neurodegenerative Diseases (CEMAND), University of Salerno, 84131 Salerno, Italy; 2 University School for Advanced Studies IUSS Pavia, Pavia, Italy; 3 IRCCS Fondazione Mondino, Pavia, Italy

**Keywords:** Cognition, Mild cognitive impairment, Language, Multiple System Atrophy

## Abstract

**Background:**

The evidence about the language performance profile of multiple system atrophy (MSA) is limited, but its definition may lead to a more comprehensive characterization of the disorder and contribute to clarify the involvement of the basal ganglia in language abilities.

**Objective:**

The objectives of the study were: (1) to evaluate the reliability of the Screening for Aphasia in NeuroDegeneration (SAND) in MSA patients; (2) compare the linguistic profiles among MSA and Parkinson’s disease (PD) patients and healthy controls (HC), and (3) assess relationships between language impairment and cognitive status and MSA motor subtypes.

**Methods and results:**

Forty patients with a diagnosis of MSA, 22 HC and 17 patients with PD were enrolled in the present study. By excluding the writing task that showed a poor acceptability, we showed that the MSA-tailored SAND Global Score is an acceptable, consistent and reliable tool to screen language disturbances in MSA. MSA patients performed worse than HC, but not than PD, in MSA-tailored SAND Global Score, repetition, reading and semantic association tasks. We did not find significant differences between MSA phenotypes. MSA patients with mild cognitive impairment-multiple domain presented worse language performances as compared to MSA patients with normal cognition and mild cognitive impairment-single domain.

**Conclusion:**

The MSA-tailored SAND Global Score is a consistent and reliable tool to screen language disturbances in MSA. Language disturbances characterize MSA patients irrespective of disease phenotype, and parallel the decline of global cognitive functions.

**Supplementary Information:**

The online version contains supplementary material available at 10.1007/s00702-021-02372-6.

## Introduction

Multiple system atrophy (MSA) is a rapidly progressive α-synucleinopathy, characterized by different combinations of progressive parkinsonism, cerebellar ataxia, autonomic failure and corticospinal impairment. Specifically, the parkinsonian variant (MSA-P) is characterized by prominent akinetic-rigid parkinsonism and the cerebellar variant (MSA-C) by progressive ataxia (Stankovic et al. 2014; Gilman et al. 2008). Cognitive impairment is common in MSA and involves primarily processing speed and attention/executive functions (Santangelo et al. 2018). Spatial planning skills, sustained attention, abstract thinking and verbal fluency are most commonly impaired (Stankovic et al. 2014). Working memory, recognition and recall of previously learned information and visuo-spatial skills can also be affected (Stankovic et al. 2014).

Speech disorders with heterogeneous features (Sachin et al. 2008) are a common clinical manifestation, occurring in 70–100% of patients with Parkinson's disease (PD) and atypical parkinsonian syndromes in medium–advanced stages (Ho et al. 1998) but also in the early stages (Rusz et al. 2011). Speech abnormalities have a significant impact on the subject's quality of life, involving an increased requirement of physical and cognitive resources during social interactions (Miller et al. 2006). The poor performance on language production tasks might be primarily the consequence of speech disorders, such as dysarthria, which is a common clinical feature of atypical parkinsonian syndromes and is related to basal ganglia pathology. Speech dysfunction in Parkinsonism may include mono-pitch, mono-loudness, imprecise consonants, inappropriate silences, all features of articulatory dysfunction and can affect speech production in terms of intelligibility, fluency, morpho-syntactic organization (Rusz et al. 2015). Moreover, the motor dysfunction of MSA could affect the language tasks that require a motor output, such as writing.

The domain of language has rarely been studied in MSA, generally with tasks largely affected by speech motor skills and overlapping with executive functions, such as the fluency tests that are considered a hybrid measure of cognitive functioning (Aita et al. 2019). To the best of our knowledge, no previous study comprehensively evaluated the language profile of MSA patients. Since the tools commonly used to evaluate language have been mostly validated for vascular patients and are not sensitive to neurodegenerative diseases, we decided to use the Screening Battery for Aphasia in NeuroDegeneration (SAND), designed for neurodegenerative diseases and already validated in the healthy Italian population.

We aimed to: (1) evaluate the reliability of the SAND in MSA patients; (2) compare the linguistic profiles among MSA and PD patients and HC, and (3) assess relationships between language impairment and cognitive status.

## Methods

### Patients

Between November 2015 and April 2019, 40 patients with a diagnosis of probable MSA, according to current criteria (Gilman et al. 2008) were enrolled at the Center for Neurodegenerative Diseases of the University of Salerno.

Additional inclusion were: (a) Italian native speaker; (b) sufficiently intelligible speech; (c) intact or corrected auditory and visual functions.

In addition, 22 healthy controls (HC) and 17 patients with diagnosis of probable idiopathic PD were also enrolled for the present study. PD were assessed ON drugs. The three groups were matched for age, education, cognitive state assessed by Mini-Mental State Evaluation (MMSE) and disease duration. HC were recruited among patients’ caregivers and had no history of neurological, psychiatric or physical illness.

All people provided informed consent. The local Ethics Committee approved the protocol.

### Clinical and cognitive evaluations

The severity of the disease was assessed by the Unified Multiple System Atrophy Rating Scale (UMSARS). The severity of dysarthria was assessed by item 2 of the UMSARS-II (Wenning et al. 2004). Since MSA patients presented a dysarthria score ranging from 1 to 3 on item 2 of the UMSARS-II, we stratified the sample into three levels (score 1, score 2, score 3).

Cognitive abilities were screened with the Montreal Cognitive Assessment (MOCA) (Santangelo et al. 2015), delayed recall scores of the Rey auditory verbal learning test (15-RAWLT), recall and copy of Rey Osterrieth figure, prose memory test, Trail Making Test (TMT), Stroop Interference Test-short version Clock design test (CDT), semantic verbal fluency test (SVF), constructional apraxia test and Benton orientation line test (BJLO) (Barletta-Rodolfi et al. 2011).

Functional autonomy was evaluated with the Instrumental Activities of Daily Life (IADL) and with the Basic Activities of Daily Life (ADL), depression and apathy with the Beck Depression Inventory II (BDI-II), using cut-off > 12 (Ghisi et al. 2006) and Apathy Evaluation Scale (AES), using cut-off > 37 (Santangelo et al. 2014).

We used the z-scores of the individual tests and a control group of thirty HC not included in the current study to classify MSA with normal cognition (MSA-NC), with Mild Cognitive Impairment (MCI) and MSA with dementia (MSA-D). Subsequently we specified the type of MCI using the following labels: MSA with MCI-single domain, MSA with MCI-multiple domain. Due to the lack of MSA-specific MCI criteria, MCI MDS criteria for Parkinson's disease (Litvan et al. 2012) were applied. As such, MCI was defined as impairment in neuropsychological tests (score less than 1.5 standard deviation) without impairment in IADL and further subdivided into single and multiple domain (Litvan et al. 2012). The diagnosis of dementia (MSA-D) was made according to the criteria of the According to the Statistical Diagnostic Manual of Psychiatry-5th Edition (DSM-5).

Following a large number of tests and possible fatigue, all patients were asked to choose whether to complete the evaluation in a one or two sessions. All patients were able to complete the assessment in one session.

### Language testing

Language was evaluated with the Screening Battery for Aphasia in NeuroDegeneration (SAND). This battery was developed given the lack of specific tools aimed at assessing language disorders in neurodegenerative diseases (Catricalà et al. 2017). It yields scores for nine sub-tests: picture naming, auditory sentence comprehension, single word comprehension, repetition of words and non-words, repetition of sentences, reading, written description, semantic association and picture description. Some sub-tests have additional sub-scores. An exhaustive description of each task of the SAND battery can be found in Catricalà et al. (Catricalà et al. 2017).

So far, the SAND has been validated in primary progressive aphasia (PPA) and progressive supranuclear palsy (PSP). Specifically, a global score was used for the identification of the psychometric properties of the battery. In PPA, the psychometric properties of SAND were calculated on a 23-task battery (Battista et al. 2005), while a tailored 19-task battery was validated in PSP (Picillo et al. 2019).

We first analyzed the reliability of the original 23-task battery in MSA patients. Such data were compared with the reliability of a MSA-tailored 17-task battery, obtained after removing the tasks with a higher percentage of missing data due to motor impairment. In particular, we removed sub-scores of the writing task (see Appendix for more details).

The language domain was also explored with two sub-tests from the Neuropsychological Examination of Aphasia battery (ENPA), that are the non-word repetition and the auditory comprehension of sentences tests (Barletta-Rodolfi et al. 2011) and with the CaGi (Catricalà et al. 2013). The SAND, ENPA and CaGi have a similar structure but different items.

### Statistical analysis

After checking for normality distribution with the Kolmogorov–Smirnov test, differences in variables between groups were computed with *χ*^2^ or the Kruskal–Wallis tests as appropriate. Pairwise comparisons were performed with Mann–Whitney *U* test.

### The psychometric properties and discriminatory power of the SAND on MSA

The acceptability and internal consistency were explored following the global score created for PPA. Subsequently, the data were compared with the reliability of a global score tailored for MSA, obtained by removing the tasks with a higher percentage of missing data. Internal consistency was evaluated by means of Cronbach’s *α*. The global score with higher internal consistency was the anchor for subsequent analysis.

Scaling assumptions referring to the correct grouping of items and the appropriateness of their summed score were checked using corrected item-total correlation for both Global Scores (standard, ≥ 0.40). Construct validity was explored with non-parametric Spearman’s correlation between the Global Score and other language testing as well as with cognitive and behavioral testing.

### Differences among MSA, PD and HC groups

We compared sub-tests of SAND and MSA-tailored SAND Global Score among MSA, PD and HC with Kruskal–Wallis test; pairwise comparisons were performed with Mann–Whitney *U* test.

### Differences within MSA group

We divided MSA patients in three groups according to the severity of dysarthria, assessed with UMSARS-II Item 2 and investigated the differences in language tests among groups by Kruskal–Wallis test. We used Spearman’s correlations to explore the relationships between dysarthria, disease duration and SAND Global Score.

We compared demographic and clinical variables according to cognitive status, namely NC, MCI, MCI-single domain/MCI-multiple domain and divided by the median score of MOCA, by Kruskal–Wallis test and, where necessary, the pair-wise comparisons were performed with Mann–Whitney *U* test. The significant demographic and clinical variables were used as covariate variables in following analysis. We performed a MANCOVA with Bootstrap method and corrected for multiple comparisons, to identify differences in language tests based on global cognitive status. Specifically, we used the significant results of Kruskal–Wallis tests as covariate variables, the SAND sub-tests and the MSA-tailored SAND Global Score as dependent variables and global cognitive status as independent variables. About global cognitive status, first, we divided patients in NC and MCI, then we refined the analysis by comparing NC, MCI-single domain and MCI-multiple domain*.* Finally, we compared the sub-scores of the SAND Global Score and the UMSARS-II Item 2 sub-scores between MSA patients divided by the median score of MOCA.

We compared sub-tests of SAND and the MSA-tailored SAND Global Score between MSA-P and MSA-C patients by means of Mann–Whitney *U* test.

Statistical analysis was performed with SPSS, Version 23.

## Results

Forty-one MSA patients were considered for the present study, but one was excluded due to unintelligible speech. The final cohort thus included 40 MSA and 17 PD patients as well as 22 HC, matched for age, education, cognitive state assessed by MMSE and disease duration. Demographics and clinical data are reported in Table 1.

**Table 1:** Demographic and clinical features of the enrolled cohort

	MSA (*N* = 40) median (IQR)	PD (*N* = 17) median (IQR)	HC (*N* = 22) median (IQR)	*p*
Age	62.00 (11.0)	64.00 (3.0)	64.00 (7.0)	0.07
Education	11.00 (7.0)	10.00 (11.0)	8.00 (7.8)	0.74
Disease duration (years)	4.00 (4.0)	6.00 (6.0)	NA	0.36
MMSE	27.00 (5.0)	28.00 (3.0)	27.50 (2.3)	0.69
UMSARS-I	22.50 (11.5)	NA	NA	NA
UMSARS-II	25.00 (10.5)	NA	NA	NA
UMSARS-IV	3.00 (2.0)	NA	NA	NA
UPDRS-III	NA	18.00 (16.0)	NA	NA

Data are in median (interquartile range = IQR), unless otherwise specified

HC, healthy controls; MMSE, Mini-mental State Examination; MSA, multiple system atrophy; NA, not applicable; PD, Parkinson’s disease; SAND, Screening for Aphasia in NeuroDegeneration; UMSARS, Unified Multiple System Atrophy Rating Scale; UPDRS, Unified Parkinson’s Disease Rating Scale

### Validation phase

The SAND tailored on PPA, composed of 23 tasks (see Appendix) and applied on MSA showed a Cronbach’s *α* of 0.696, but there were 10% missing data (see Appendix-Table S1). Considering that the Cronbach’s *α* value was suboptimal for internal consistency, we removed the items presenting poor acceptability (writing task) and obtained an MSA-tailored Global Score composed of 17 tasks. The Cronbach’s *α* of SAND tailored on MSA was equal to 0.728, indicating high-level internal consistency. Therefore, we used the MSA-tailored SAND Global Score for the following analyses (see Appendix-Table S2). Neither ceiling nor floor effects were observed for the MSA-tailored SAND Global Score (lowest possible score = 0, 18%; highest possible score = 12, 2.5%). Skewness of the MSA-tailored SAND Global Score was 1.106. All the MSA-tailored SAND Global Score items presented excellent acceptability as there were no missing data and 100% of data were computable. By removing additional items, no further improvement of Cronbach’s *α* was detected. Spearman’s correlation confirmed convergent validity of the single tasks included in the MSA-tailored SAND Global Score, showing a significant correlation with language tests (see Appendix-Table S3). As for the other cognitive tests, moderate correlation was present with measures of global cognition as the MMSE and the MOCA, but no correlation was shown with memory test and apathy and depression scores (see Appendix-Table S4).

### Language differences among MSA, PD and HC

MSA patients performed worse in total MSA-tailored SAND Global Score, in repetition of words/no-words, repetition of predictable and unpredictable sentences, reading of words, semantic association and Number of phonological errors/number of words, and picture description as compared to HC (*p* < 0.01). There were no significant differences between MSA and PD patients PD performed better in number of nouns/number of total words and number of repaired sequences/number of words in picture description task than HC (*p* < 0.01) (Table 2).

**Table 2:** Comparisons of MSA-tailored SAND Global Score and SAND sub-test scores among MSA, PD and HC

	MSA (*N* = 40) median (IQR)	PD (*N* = 17) median (IQR)	HC (*N* = 22) median (IQR)	*p*
MSA-tailored SAND Global Score	2.00 (4.0)^a^	1.00 (2.0)	1.00 (2.0)^a^	**0.01**
Picture naming total	12.00 (2.3)	13.00 (2.0)	13.00 (2.0)	**0.04**
Auditory sentence comprehension	8.00 (1.0)	8.00 (0.0)	8.00 (0.5)	0.19
Single word comprehension-total	12.00 (1.0)	12.00 (0.5)	12.00 (2.0)	0.44
Words/no-words repetition-total	7.00 (1.5)^a^	9.00 (1.5)	9.00 (2.0)^a^	**< 0.01**
Sentence repetition-total	3.00 (2.5)^a^	4.00 (2.5)	5.00 (1.5)^a^	**< 0.01**
Reading-total	14.00 (4.0)^a^	15.00 (1.5)	16.00 (1.0)^a^	**< 0.01**
Writing, information units	3.00 (2.5)	4.00 (4.0)	5.00 (1.0)	**< 0.01**
Semantic association	3.00 (1.0)^a^	4.00 (1.0)	4.00 (1.0)^a^	0.54
Picture description, information units	5.00 (3.5)	6.00 (4.0)	5.00 (3.0)	0.09
Number words—picture description	61.00 (55.0)	91.00 (68.0)	83.00 (62.0)	**0.02**
Number of nouns/number of total words—picture description	0.29 (0.1)	0.25 (0.1)^c^	0.29 (0.1)^c^	**0.02**
Number of verbs/number of total words—picture description	0.15 (0.1)	0.14 (0.1)	0.17 (0.1)	0.39
Total number of syntactic structures—picture description	8.00 (6.5)	10.00 (8.0)	10.00 (6.5)	**0.02**
Number of subordinates/total number of syntactic structures—picture description	0.16 (0.5)	0.30(0.2)	0.20 (0.6)	0.56
Number of repaired sequences/number of words—picture description	0.00 (0.0)	0.01 (0.1)^c^	0.00 (0.0)^c^	**< 0.01**
Number of phonological errors/number of words—picture description	0.00 (0.0)^a^	0.00 (0.0)	0.00 (0.0)^a^	**< 0.01**
Lexical-semantic errors/number of words—picture description	0.00 (0.0)	0.00 (0.0)	0.00 (0.0)	**0.02**

Data are in median (interquartile range = IQR), unless otherwise specified. Statistically significant differences are indicated in bold

HC, healthy controls; MSA, multiple system atrophy; PD, Parkinson’s disease; SAND, Screening for Aphasia in NeuroDegeneration

^a^MSA versus HC (corrected *p* ≤ 0.01)

^b^MSA versus PD (corrected *p* ≤ 0.01)

^c^PD vs HC (corrected *p* ≤ 0.01)

### Language differences within MSA patients

According to the cognitive status, we found 25 patients with NC and 15 MCI. By Kruskal–Wallis test, we found that groups had significantly different age (*p* < 0.01) and UMSARS-I scores (*p* < 0.05) but did not differ as for education, UMSARS-II and UMSARS-III scores (*p* > 0.05). By MANCOVA, using age and UMSARS-I scores as covariates, we found that MSA with MCI showed worse scores than MSA-NC in MSA-tailored SAND Global Score, naming of living and non-living, words and not words repetition and Semantic associations (*p* ≤ 0.01) (Table 3).

**Table 3:** Comparisons of MSA-tailored SAND Global Score and SAND sub-test scores between MSA-NC, MSA with MCI, by MANCOVA, corrected for multiple comparisons and using age, education and UMSARS-I as covariate variables

	MSA-NC (*N* = 25) median (IQR)	MSA-MCI (*N* = 15) median (IQR)	*p*
MSA-tailored SAND Global Score	1.50 (2.8)^a^	5.00 (5.0)^a^	**< 0.01**
Picture naming total	13.00 (1.5)^a^	11.00 (4.0)^a^	**< 0.01**
Auditory sentence comprehension	8.00 (1.0)	7.00 (2.0)	0.02
Single word comprehension-total	12.00 (0.0)	11.00 (2.5)	0.20
Words/no-words repetition-total	8.00 (2.0)^a^	7.00 (2.0)^a^	**< 0.01**
Sentence repetition-total	3.50 (2.0)	3.00 (1.0)	0.06
Reading-total	15.00 (3.0)	13.00 (4.5)	0.77
Writing, information units	4.50 (2.0)	3.00 (3.5)	0.44
Semantic association	4.00 (1.0)^a^	3.00 (1.5)^a^	**< 0.01**
Picture description, information units	5.00 (4.0)	5.00 (4.0)	0.48
Number words—picture description	65.5 (60.0)	42.00 (54.5)	0.68
Number of nouns/number of total words—picture description	0.28 (0.1)	0.30 (0.1)	0.51
Number of verbs/number of total words—picture description	0.15 (0.1)	0.13 (0.1)	0.25
Total number of syntactic structures—picture description	8.0 (5.0)	6.00 (6.0)	0.94
Number of subordinates/total number of syntactic structures—picture description	0.31 (0.5)	0.00 (0.3)	0.15
Number of repaired sequences/number of words—picture description	0.00 (0.0)	0.00 (0.0)	0.23
Number of phonological errors/number of words—picture description	0.00 (0.0)	0.00 (0.0)	0.54
Lexical-semantic errors/number of words—picture description	0.00 (0.0)	0.00 (0.0)	0.28

Data are in median (Interquartile range = IQR), unless otherwise specified. Statistically significant differences are indicated in bold

MCI, mild cognitive impairment; MSA, multiple system atrophy; NC, normal cognition; SAND, Screening for Aphasia in NeuroDegeneration

^a^MSA with NC versus MSA with MCI (corrected *p* ≤ 0.01)

In the second step, we found that 25 patients had NC, 7 MCI-single domain and 8 MCI-multiple domain. By Kruskal–Wallis test and Mann–Whitney *U* test, we found that patients with MCI-multiple domain had a significantly older age and lower education than MSA-NC. Using age and education as covariate variables, the MANCOVA showed that MSA with MCI-multiple domain had worse scores than MSA-NC in MSA-tailored SAND Global Score, naming of living and non-living and Semantic associations (*p* ≤ 0.01) (Table 4).

**Table 4:** Comparisons of MSA-tailored SAND Global Score and SAND sub-test scores among MSA-NC, MSA with MCI-single domain and MSA with MCI-multiple domain, by MANCOVA and post hoc analyses, corrected for multiple comparisons and using age and education as covariate variables

Test	NC (*N* = 25) median (IQR)	MCI-single domain (*N* = 7) median (IQR)	MCI-multiple domain (*N* = 8) median (IQR)	*p*
MSA-tailored SAND Global Score	1.00 (2.5)^a^	2.00 (4)	6.50 (3.8)^a^	**< 0.01**
Picture naming—total	13.00 (1.6)^a^	11.00 (3.5)	9.50 (4.0)^a^	**< 0.01**
Auditory sentence comprehension	8.00 (1.0)	7.00 (1.5)	7.00 (4.0)	0.04
Single word comprehension—total	12.00 (0.3)	11.50 (1.0)	11.00 (4.0)	0.27
Words/no-words repetition—total	8.00 (2.0)	7.00 (1.5)	6.50 (2.0)	**0.03**
Sentence repetition—total	3.00 (2.0)	3.00 (0.8)	2.00 (1.0)	0.16
Reading—total	15.00 (3.3)	14.00 (2.8)	12.00 (6.0)	0.71
Writing, information units	3.50 (2.0)	2.50 (2.5)	3.00 (4.0)	0.23
Semantic associations	4.00 (1.0)^a^	3.00 (2.3)	2.00 (2.0)^a^	**0.01**
Picture description, information units	5.00 (4.0)	5.50 (4.0)	5.00 (5.0)	0.97
Number words—picture description	64.5 (59.5)	57.5 (131.8)	38.0 (34.0)	0.53
Number of nouns/number of total words—picture description	0.28 (0.1)	0.38 (0.3)	0.30 (0.1)	0.35
Number of verbs/number of total words—picture description	0.15 (0.1)	0.17 (0.2)	0.11(0.1)	0.37
Total number of syntactic structures—picture description	8.00 (5.5)	7.00 (13.3)	4.00 (7.0)	0.18
Number of subordinates/total number of syntactic structures—picture description	0.31 (0.5)	0.11 (0.2)	0.00 (0.5)	0.26
Number of repaired sequences/number of words—picture description	0.00 (0.0)	0.00 (0.1)	0.00 (0.0)	0.09
Number of phonological errors/number of words—picture description	0.00 (0.0)	0.00 (0.0)	0.00 (0.0)	0.32
Lexical-semantic errors/number of words—picture description	0.00 (0.0)	0.00 (0.0)	0.00 (0.0)	0.18

Data are in median (interquartile range = IQR), unless otherwise specified

MCI, mild cognitive impairment; MSA, multiple system atrophy; NC, normal cognition; SAND, Screening for Aphasia in NeuroDegeneration

^a^MSA-NC vs MSA with MCI-multiple domain (corrected *p* ≤ 0.01)

^b^MSA-NC vs MSA with MCI-single domain (corrected *p* ≤ 0.01)

^c^MSA with MCI- single domain versus MSA with MCI-multiple domain (corrected *p* ≤ 0.01)

Patients with MOCA lower than the median score of 20 (*n* = 18/40) were less educated, higher age and UMSARS-II than patients with higher score. No significant differences were found in scores between groups, using age, education and UMSARS-II as covariate.

Dividing MSA patients in 3 groups according to the severity of dysarthria, we did not find significant differences among groups in MSA-tailored SAND Global Score (*p* = 0.94); there was no significant correlation between MSA-tailored SAND Global Score and both dysarthria (*p* = 0.14) and disease duration (*p* = 0.21).

By comparing sub-tests of SAND and MSA-tailored SAND Global Score between MSA-P and MSA-C patients, MSA-C (*n* = 20) performed better than MSA-P (*n* = 20) in the number of repaired sequences/number of words in picture description (*U* = 110.50, *p* < 0.01). We also compared MSA-P with PD and results are shown in Appendix-Table S5.

## Discussion

In this study, we evaluated an often overlooked aspect of cognitive dysfunction in movement disorders, namely language impairment. Indeed, a growing number of studies, especially regarding idiopathic PD, reported significant difficulties with language tasks, including understanding of sentences, verbal fluency and naming (Picillo et al. 2019; Catricalà et al. 2013, 2019). Generally, language alterations have also been reported in patients with lesions of the basal ganglia following stroke (Auclair-Ouellet et al. 2017). Language involves different cognitive processes and therefore language tasks can provide rich information on the cognitive status of patients with neurodegenerative disorders (Catricalà et al. 2019). In fact, recognizing language disorders and their relationship with motor disability can be useful both clinically and theoretically (Pickett et al. 1998).

### Validation phase

We found that the MSA-tailored SAND Global Score, composed of 17 tasks, is an acceptable, reliable and easily applicable tool to explore language profile in MSA patients. By removing sub-scores with high proportion of missing values, we obtained a significant improvement in consistency and acceptability comparable with the original SAND Global Score and PSP-tailored SAND (Battista et al. 2005; Picillo et al. 2019).

As a matter of fact, unlike patients with PPA, MSA patients have motor features possibly impacting performance on the writing task, such as dystonia and bradykinesia. The MSA-tailored SAND Global Score overcame such limitation showing high acceptability, since data were computable for 100% and the percentage of missing values was 0% for all items. The excellent acceptability in MSA is confirmed by the absence of both ceiling and floor effects. Furthermore, the internal consistency of MSA-tailored SAND Global Score is high, suggesting a coherent representation of all the language functions screened. As for convergent construct validity, each task of the MSA-tailored SAND Global Score showed significant moderate correlation values with other corresponding language testing. Furthermore, the MSA-tailored SAND Global Score showed moderate correlation with measures of global cognition as well as with cognitive tests exploring attention-executive and visuo-spatial domains. Our results suggest that an objective linguistic assessment may provide a widely applicable screening tool for better characterization of MSA patients. Dysarthria, as measured by the UMSARS-II item 2, was not related with MSA-tailored SAND Global Score, indicating that the SAND battery is useful to investigate different aspects of language independent of articulation impairment. Having a reliable screening language test for MSA patients can be helpful in studying this function without the limitations of previous approaches using tests overlapping with executive functions (Aita et al. 2019), single tests that measured only one aspect of language or batteries unsuitable for patients with motor deficits (Leisman et al. 2016).

### Language differences among MSA, PD and HC

MSA performed worse in MSA-tailored SAND Global Score, naming, repetition of words/non-words, repetition of sentences, reading of words, total number of syntactic structures in picture description and information units of writing sub-tests as compared to HC. Our results on the naming test are in line with the literature reports of impaired naming as a frequent feature of many different neurological disorders (Miceli et al. 1994). The naming performance may also depend on the integrity of non-linguistic abilities, supporting the hypothesis that impaired language abilities in MSA can be interpreted within an embodied cognition framework (Archibald and Joanisse 2009). Our results on repetition task could be explained by the dys-executive deficits commonly found in MSA, and specifically by altered interactions among working memory, processing speed and language domain (Miceli et al. 1994; Archibald and Joanisse 2009). Therefore, we can conclude that language deficits in MSA are not limited to speech problems, but also to alterations of executive function and of “embodied” aspects of language.

MSA did not perform worse than PD patients in the MSA-tailored SAND global score and sub-scores. This result is consistent with a previous study that found no differences in ENPA results between MSA and PD patients (Santangelo et al. 2018). In another previous study, patients with MSA, PD and Lewy Body Dementia (DLB) performed equally well on simple tests of sentence repetition, object naming and lexical fluency, but both DLB and MSA subjects showed decreased semantic fluency as compared to PD subjects (Antzoulatos and Miller 2011). After using a more extensive language battery specific for neurodegenerative diseases and including more patients than the previous study, we can confirm that the language domain do not significantly differentiate MSA from PD patients matched for disease duration.

Surprisingly, PD patients performed better in number of nouns/number of total words and number of repaired sequences/number of words in picture description task as compared to HC; however, we believe that this may be due to increased attention reported by PD patients to the test, since no other difference was found in this extended battery.

### Language differences in MSA patients according to global cognitive state and phenotypes

As for the relationship between language and cognitive status, we detected worse language performance in MSA patients with each type of MCI compared to NC. MCI-multiple domain showed a worse performance in total MSA-tailored SAND Global Score, naming and semantic association as compared to MSA-NC. These results are partially consistent with the impairment in naming tests reported in 6 MSA patients with dementia as opposed to 9 MSA patients without dementia in a previous study (Kao et al. 2009). Furthermore, in this study, there was no significant difference on the phonemic fluency test between MSA with and without dementia (Kao et al. 2009). Indeed, it has been reported that also in PD, global cognitive profile may influence naming performances (Kim et al. 2013).

Both analyses showed a prominent involvement of semantic domain, mainly affecting naming and semantic memory skills, in MSA patients with MCI.

We did not find differences between MSA-C and MSA-P phenotypes in MSA-tailored SAND Global Score. As for language differences between MSA-P and MSA-C, only differences in fluency tests have been reported in literature (Bocanegra et al. 2015), but it is known that fluency tests can be affected by speech motor problems and are usually used to assess executive functions, that are the most compromised in movement disorders (Aita et al. 2019). Therefore, we suggest that the language profile in MSA patients does not change according to the motor phenotype, but is only affected by level of patients’ cognitive impairment.

## Conclusion

We first looked at the properties of a new language screening test in MSA patients and were able to comprehensively assess a cognitive domain that had previously only been studied by semantic and phonemic fluency and naming tasks. By applying this new tool in MSA, our study provides new evidence to support the notion of a language–action relationship, which would depend on the integration between cortical and subcortical areas (Barcelos et al. 2018) and, although further investigation is needed, helps to clarify that basal ganglia could play an important role in language function, since degenerative disorders that primarily affect them impair speech at a clinically important and measurable level.

## Supplementary Information

Below is the link to the electronic supplementary material.

Supplementary file1 (DOC 82 kb)

## Abbreviations

15-RAWLT: Rey’s auditory 15-word learning test
ADL: Based activities of daily life
AES: Apathy Evaluation Scale
BDI-II: Beck Depression Inventory II
BJLO: Benton’s Judgment of Line Orientation
CDT: Clock drawing test
DLB: Dementia with Lewy body
DSM-5th: Statistical Diagnostic Manual of Psychiatry-5th Edition
ENPA: Neuropsychological Examination of Aphasia battery
H&Y: Hoehn and Yahr
HC: Healthy controls
IADL: Instrumental activities of daily life
MCI: Mild cognitive impairment
MDS: Movement Disorders Society
MMSE: Mini-Mental State Examination
MOCA: Montreal Cognitive Assessment battery
MSA: Multiple system atrophy
MSA-C: Multiple system atrophy with predominantly cerebellar ataxia
MSA-D: Multiple system atrophy with dementia
MSA-NC: Multiple system atrophy with normal cognition
MSA-P: Multiple system atrophy with predominantly parkinsonism
NA: Not applicable
*p*: *p* Value
PD: Parkinson disease
PPA: Primary progressive aphasia
PSP: Progressive supranuclear palsy
SAND: Screening for Aphasia in NeuroDegeneration
SCWT: Stroop Color-Word Test
SPSS: Statistical Package for Social Science
SVP: Semantic verbal fluency
TMT: Trial Making Test
TMT-A: Part A of Trail Making Test
UMSARS: Unified Multiple System Atrophy Rating Scale
UPDRS: Unified Parkinson’s Disease Rating Scale
