# Supplementary material for: The language profile in multiple system atrophy: an exploratory study
Source: J Neural Transm (Vienna). 2021 Jul 3;128(8):1195–203. doi: 10.1007/s00702-021-02372-6 (PMC8322009; doi:10.1007/s00702-021-02372-6)
Supplement: Supplementary file 1 — Supplementary file1 (DOC 82 kb) [file 702_2021_2372_MOESM1_ESM.doc]

**Appendix**

## Supplementary methods

The SAND provides a brief comprehensive language assessment tailored for patients with Primary Progressive Aphasia (PPA), including: (1) Picture naming: The subject is asked to name 14 black and white object drawings; (2) Sentence comprehension: The subject is asked to choose which of two pictures matches the meaning of the sentence read by the examiner. The sentences include two short active, two short passive, two coordinates and two embedded structures; (3) Word comprehension: The subject is asked to point at the target among four object pictures in response to a spoken word; (4) Repetition: The subject is asked to repeat words and non-words read by the examiner; (5) Sentence repetition: The subject is asked to repeat the sentences read by the examiner; (6 ) Reading: The subject is asked to read regular and irregular words and non-words; (7) Semantic association: the subject is asked to point at the two semantically related images out of three; (8) Writing: The subject is asked to describe how to brush their teeth; (9) Picture description task: The subject is asked to describe a complex picture [16, 17].

The entire battery is short and can be administered to MSA patients in less than 20 minutes. For each of the 9 subtests a score can be computed. In addition, picture description and written description analysis yields additional sub-scores, resulting in a total of 23 task-related scores [16, 17].

Hence, the SAND Global Score including the 23 task-related scores was computed according with a previously described three steps process: (1) The raw scores were adjusted by adding or subtracting the influence of age, sex, and education and corrected using available normative data [16]; (2) Corrected scores were compared with the corresponding cutoff values obtained from HC; (3) The sum of the twenty-three dichotomous variables (1=pathological, 0=normal) represented the SAND Global Score, with higher scores indicating more severe impairment (SAND global score range:0–23) [17].

The Global score of SAND composed of 23 tasks showed a suboptimal acceptability in MSA patients, due to a high proportion of missing data in the writing tasks. More than half of MSA patients with missing data refused or were unable to complete such tasks; this was generally due to hand dystonia and/or bradykinesia.

***Supplementary data -Table S1:*** ***Valid and missing data of SAND battery: comparison between the writing and all the remaining SAND tasks.***

|  | **Writing task** | **All the remaining SAND tasks** |
| --- | --- | --- |
| **Valid data** | 90% | 100% |
| **Missing data** | 10% | 0 % |

Therefore, following the three steps process as noted above, an MSA-tailored SAND Global Score was created, reducing the impact of the writing sub-scores. The MSA-tailored SAND Global Score ranges from 0 to 17, with higher scores indicating greater impairment.

By reducing the items of writing tasks the acceptability of the SAND battery presented an improvement (see Results).

Additional inclusion criteria for the present study were: (a) Italian native speaker status; (b) sufficiently intelligible speech such that the intended target could be determined for the majority of words; (c) intact or corrected auditory and visual functions; (d) successful completion of the language testing.

Moreover, in order to reinforce the our chose to use 17 tasks for MSA-tailored SAND Global Score, we calculated also in PD sample the Cronbach’ s alpha that was equal to 0.761, an optimal value.

**Supplementary data -Table S2:** **The comparison of tasks used for SAND reliability analysis in PPA and MSA populations**

| **PPA- tailored SAND Global score (0-23)**  **(Battista et al., 2018)** | **MSA-tailored SAND Global score (0-17)**  **(Our proposal)** |
| --- | --- |
| **A)**Naming  1)Total  **B**)Sentence comprehension  **C**)Single word comprehension  1)Total  **D**)Repetition  1)Total  **E**)Sentence repetition  1)Total  **F**)Reading  1)Total  **G**)Semantic associations  **H**)Writing  1)Information units  2)Total words  3)Nouns/total words  4)Verbs/total words  5)sentences  6)Orthographic errors  7)semantic errors  **I**)Picture description  1)Informative units  2)Number of words  3)Nouns/words  4)Verbs/words  5)Repaired sequences/number of words  6)Sentences  7)Subordinate/sentences  8)Phonological errors/number of words  9)Semantic errors/number of words | **A)**Naming  1)Total  **B**)Sentence comprehension  **C**)Single word comprehension  1)Total  **D**)Repetition  1)Total  **E**)Sentence repetition  1)Total  **F**)Reading  1)Total  **G**)Semantic associations  **H**)Writing  1)Information units  **I**)Picture description  1)Informative units  2)Number of words  3)Nouns/words  4)Verbs/words  5)Repaired sequences/number of words  6)Sentences  7)Subordinate/sentences  8)Phonological errors/number of words  9)Semantic errors/number of words |

**Abbreviations:** MSA, multiple system atrophy; PPA: Primary Progressive Aphasia

**Supplementary data -Table S3**: **Spearman’s correlation between single tasks of the MSA-tailored SAND Global Score and other language tests**

| **SAND Task** | **Language tests** | **Spearman’s correlation** | **P** |
| --- | --- | --- | --- |
| **Naming** | *Category fluency*  *CaGi naming* | 0.474  0.611 | **0.002**  **0.002** |
| **Word comprehension** | *Category fluency*  *Auditory sentence comprehension (ENPA)* | 0.682  0.646 | **< 0.001**  0.004 |
| **Sentence comprehension** | *Auditory sentence comprehension (ENPA)* | 0.280 | 0.085 |
| **Words/no-words repetition** | *Category fluency* | 0.330 | 0.038 |
| **Sentence repetition** | *Sentence repetition (ENPA)*  *Buccofacial apraxia test* | 0.500  0.395 | **< 0.001**  0.046 |
| **Reading** | *Word repetition (ENPA)*  *Sentence repetition (ENPA)*  *Auditory sentence comprehension (ENPA)*  *CaGi naming* | 0.443  0.419  0.507  0.467 | 0.005  0.009  0.032  0.025 |
| **Writing I.U.** | *Category fluency*  *No-word repetition (ENPA)*  *Sentence repetition (ENPA)*  *Auditory sentence comprehension (ENPA)* | 0.367  0.459  0.519  0.436 | 0.027  0.005  **< 0.001**  0.009 |
| **Semantic association** | *Category fluency*  *Auditory sentence comprehension (ENPA)*  *CaGi naming* | 0.455  0.444  0.443 | **0.003**  0.005  0.034 |
| **Picture description I.U.** | *Sentence repetition (ENPA)*  *Auditory sentence comprehension (ENPA)* | 0.430  0.477 | 0.006  **0.002** |

Significance threshold corrected for multiple comparisons p= 0.002; significant differences are highlighted in bold.

Abbreviations: ENPA, Esame Neuropsicologico dell’Afasia; I.U., Informative Units; MSA, multiple system atrophy; SAND, Screening for Aphasia in NeuroDegeneration.

***Supplementary data -Table S4: Spearman’s correlation between the MSA-tailored Global Score MSA and non-language tests.***

|  | **Spearman’s correlation** | **P** |
| --- | --- | --- |
| ***Screening of global cognition*** | | |
| **MMSE** | -0.553 | **< 0.001** |
| **MoCA** | -0.472 | **0.002** |
| *Memory* | | |
| **RAVLT immediate** | -0.298 | 0.062 |
| **Prosa test** | -0.153 | 0.346 |
| *Visuo-spatial functioning* | | |
| **Constructional apraxia** | -0.445 | 0.005 |
| **BJLO** | -0.585 | **< 0.001** |
| *Attention-executive functions* | | |
| **CDT** | -0.324 | 0.047 |
| **TMT-A** | 0.519 | **< 0.001** |
| *Behavioral tests* | | |
| **BDI-II** | 0.294 | 0.086 |
| **AES** | 0.162 | 0.033 |

Significance threshold corrected for multiple comparisons ≤ 0.005.

Abbreviations: AES, Apathy Evaluation Scale; BDI-II, Beck Depression Inventory II; BJLO, Benton’s Judgment of Line Orientation; CDT, Clock Drawing test; MMSE, Mini-Mental State Examinaton; MoCA, Montreal Cognitive Assessment battery; RAVLT, Rey’s auditory 15-word learning test; TMT-A, Trial Making Test.

***Supplementary data -Table S5: Comparisons of MSA-tailored SAND Global Score and SAND sub-test scores between MSA-P and PD patients.***

|  | **MSA-P**  **N= 20**  **median (IQR)** | **PD**  **N= 17**  **median (IQR)** | **U** | **p** |
| --- | --- | --- | --- | --- |
| **Age** | 62.00(11.5) | 64 (3) | 122 | 0.149 |
| **Education** | 8.00(6.0) | 10 (11) | 152 | 0.578 |
| **Duration** | 5.0 (5.0) | 6 (6) | 82 | 0.585 |
| **MSA-tailored SAND Global Score** | 2.5 (4.0) | 1 (2) | 116.5 | 0.127 |
| **Picture Naming total** | 13.00(1.50) | 13.00 (2.0) | 124 | 0.152 |
| **Auditory Sentence comprehension** | 7.00(1.0) | 8.00 (0.00) | 112 | **0.033** |
| **Single Word comprehension** | 12.00(1.0) | 12.00 (0.50) | 140 | 0.269 |
| **Words/non words repetition** | 7.00(1.50) | 9.00 (1.50) | 118 | 0.104 |
| **Sentence repetition** | 4.00(2.5) | 4.00 (2.50) | 128 | 0.193 |
| **Reading** | 15.00(3.50) | 15.00 (1.50) | 123.5 | 0.142 |
| **Writing I.U.** | 3.00(2.0) | 4.00 (4.0) | 134 | 0.713 |
| **Semantic association** | 3.00 (1.8) | 4.00 (1.0) | 140 | 0.322 |
| **Picture description I.U.** | 6.00(5.50) | 6.00 (4.0) | 155.5 | 0.654 |
| Number words | 84.00(77.50) | 91.00 (68.00) | 109.000 | 0.063 |
| Number of nouns/number of total words | 0.28(0.09) | 0.25 (0.07) | 133.500 | 0.265 |
| Number of verbs/number of total words | 0.14(0.09) | 0.14 (0.07) | 167.500 | 0.939 |
| Total number of syntactic structures | 8.00(8.0) | 10.00 (8.00) | 121.500 | 0.138 |
| Number of subordinates/total number of syntactic structures | 0.17(0.52) | 0.30(0.24) | 140.000 | 0.357 |
| Number of repaired sequences/number of words | 0.01(0.02) | 0.01 (0.05) | 146.500 | 0.461 |
| Number of phonological errors/number of words | 0.00(0.01) | 0.00 (0.0) | 145.500 | 0.340 |
| Lexico-semantic errors/number of words | 0.00(0.01) | 0.00 (0.00) | 139.000 | 0.268 |

Significant differences are highlighted in bold.

Abbreviations: I.U., information units, MSA-P, multiple system atrophy with predominantly parkinsonism; PD, Parkinson Disease; SAND, Screening for Aphasia in NeuroDegeneration.
